# On the Biomimetic Design of Agile-Robot Legs

**DOI:** 10.3390/s111211305

**Published:** 2011-11-28

**Authors:** Elena Garcia, Juan Carlos Arevalo, Gustavo Muñoz, Pablo Gonzalez-de-Santos

**Affiliations:** Centre for Automation and Robotics, CSIC-UPM, La Poveda 28500, Madrid, Spain; E-Mails: juan.arevalo@car.upm-csic.es (J.C.A.); gustavo.munoz@car.upm-csic.es (G.M.); pablo.gonzalez@csic.es (P.G.-S.)

**Keywords:** legged robots, agile quadrupeds, biomimetic design, new actuators for robots

## Abstract

The development of functional legged robots has encountered its limits in human-made actuation technology. This paper describes research on the biomimetic design of legs for agile quadrupeds. A biomimetic leg concept that extracts key principles from horse legs which are responsible for the agile and powerful locomotion of these animals is presented. The proposed biomimetic leg model defines the effective leg length, leg kinematics, limb mass distribution, actuator power, and elastic energy recovery as determinants of agile locomotion, and values for these five key elements are given. The transfer of the extracted principles to technological instantiations is analyzed in detail, considering the availability of current materials, structures and actuators. A real leg prototype has been developed following the biomimetic leg concept proposed. The actuation system is based on the hybrid use of series elasticity and magneto-rheological dampers which provides variable compliance for natural motion. From the experimental evaluation of this prototype, conclusions on the current technological barriers to achieve real functional legged robots to walk dynamically in agile locomotion are presented.

## 1. State-of-the-Art Agile Legged Locomotion

### 1.1. Machines

Legged locomotion is attracting the interest of researchers of a broad range of areas. Engineers, biologists and neurologists are all pooling their knowledge for the success of a legged locomotion device [1]. The major impetus for this technology is coming from the government of the United States of America, which has given a boost to the topic through a significant number of programs sponsored by the Defense Advanced Research Projects Agency (DARPA) in the last ten years [2]. Some of these programs started in 2001 and ended recently while others are still open. The more relevant examples are the Learning Locomotion Program [3]; The BigDog Program [4]; The Exoskeletons for Human Performance Augmentation Program [5] and the more recent Legged Squad Support System Program [6]. However, despite the strong impulse in the research and the forecasted demand for these robots [7], very few advances in real applications exist. The challenges of autonomy and the large power-to-weight ratio demanded for the actuators make conventional actuation and control technology, inherited from industrial robotics, inadequate for the most promising field and service applications of legged locomotion which exhibit significant expected impact on the future society [7]. The HADE (Hybrid Actuator Development) project [8] developed at the Centre for Automation and Robotics at the Spanish National Research Council (CSIC) aims primarily at solving this problem by establishing a new line of research focused on specific actuation and control technologies for the new generation of legged robots: Agile-locomotion robots.

Quadruped robots, emulating their biological counterparts, are the best choice for field missions in a natural environment. However, it is well known that current legged-locomotion devices feature high complexity and very low speed particularly if high payloads have to be transported, and are far from reaching the performance of biological quadrupeds in natural environments.

Table 1 lists most relevant quadruped robots developed in the last 12 years, showing their payload-to-weight ratios and maximum dimensionless forward speed, which has been computed from robot dimensions and speed published by the robot’s authors as $\hat{u}=\sqrt{\mathrm{FR}}$, where FR is the Froude number, obtained from [18]:

(1) $$\mathrm{FR}=\frac{v^{2}}{\mathrm{gL}}$$

being *v*, the forward speed; *g* = 9.81 *m s*^−2^; and *L*, the characteristic leg length.

A legged vehicle designed to perform in a natural terrain should be provided with optimum performance against mobility, payload, and endurance. Such specifications were imposed by DARPA for an Agile Ground Vehicle in the UGCV program [19]. A similar denomination is here used for a legged ground vehicle, which we call “Agile” if it is able to reach a dimensionless speed of *u* = 0.54 and features a payload-to-weight ratio larger than 1, to be comparable with biological quadrupeds. Besides, a quadruped robot to perform using high-speed gaits (*i.e.*, trot and gallop) must make a thrust to the ground yielding dynamic impact loads that could exceed three times the static load on the supporting leg [20]. In a trot or in a dynamic walk, where two legs thrust the ground simultaneously, both legs share the body weight and payload, so the static load on each leg is one half the robot’s weight and added payload. Therefore, in a trot gait dynamic loads on each leg can reach 1.5 times the robot’s weight and payload. For a dynamic walk, dynamic impact loads at each leg approximately equal the robot’s weight and payload. Therefore, the structural design of an agile robot leg should make sure a load capacity-to-robot’s weight ratio of 1–1.5 depending on the envisaged gait.

**Figure 1.**
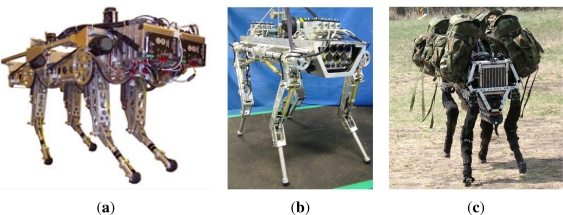
State-of-the-art agile robots: **(a)** KOLT, joint project by Stanford University and The Ohio State University, image courtesy of Prof. Waldron; **(b)** HyQ, image courtesy of the Italian Institute of Technology; **(c)** BigDog, image courtesy of Boston Dynamics.

Biological quadrupeds transition from walking to running gaits at a dimensionless speed between 0.54 to 0.7. Concretely, horses transition from walk to trot at *û* = 0.59 [21]. As exceeding this dimensionless speed requires the quadruped to run (trot or gallop) and some complex terrains could impede the use of those high-speed gaits, we have considered the dimensionless speed of 0.54 as the lower speed limit for a legged robot to be considered agile. By having a look to Table 1 it is noticed that very few quadrupeds achieve agile locomotion performance, because those robots featuring dimensionless speed above 0.54 have almost negligible payload-to-weight ratio. Although some research labs are working in this direction (Stanford University [22], Italian Institute of Technology [23]), the only existing robot reaching those targets is BigDog [4], a quadruped under development at Boston Dynamics (USA) (see Figure 1). The BigDog project is sponsored by DARPA, the US Marine Corps and the US Army. The goal of the BigDog Project is to build a class of agile unmanned ground vehicles with rough-terrain mobility superior to existing wheeled and tracked vehicles. The BigDog robots currently built “have taken the steps toward these goals, though there remains significant work to be done” [4]. Unfortunately, the ins and outs of the technology underlying BigDog are not available to the research community.

### 1.2. Actuation Systems

Supplying power to a high-speed legged machine using currently available actuation technology is a challenge. This is particularly true if the machine is expected to be energetically autonomous. In dynamic locomotion, the load experienced by each leg is at least three times the static load on that leg, and it may be much more for running gaits. The cost of building a structure and actuation system capable of providing the performance needed for the dynamic locomotion of a mid- to large-sized machine is prohibitive, even without considering payload.

Considering the mammalian muscle as a reference, direct measurements of muscle function have yielded insight into the versatile way muscles operate. It has been discovered that muscles act as motors, brakes, springs, dampers and struts [24]. The multifunctionality of natural muscle distinguishes it from any human-made actuator and it may hold the key to the success of legged locomotion. In many biological tissues it is hard to distinguish between material and structure. The use of viscoelastic materials can give the robot the spring-mass energy-cycling capacities of legged-animal locomotion, which also reduces the computational complexity of the control. Viscoelastic materials greatly simplify the mechanics of the robot, serving simultaneously as shock absorbers, springs and complete joints.

The spring-mass energy-cycling capabilities can play a key role in the dynamic locomotion of a legged vehicle. Kinetic energy can only be put into the system when the foot is on the ground. It is necessary to keep the mechanical energy in the system by using internal energy storage, that is, compliant actuation. Moreover, the leg is a mechanical oscillator, and it is energetically expensive to drive it at a frequency significantly different to its natural frequency [20]. Any means to modify the natural frequency on the leg would help to make it oscillate at different frequencies with optimal energy expenditure. Thus, inherent adaptable compliance is required [25]. Therefore, to efficiently run a dynamic legged vehicle, high power-density high force-density fast actuators with adaptable compliance are required. Added to this, energetic autonomy is expected. It is evident that these requirements are not met together by conventional technology [26].

HADE is a long-term project [8] aimed at designing energy efficient, large power-to-weight ratio actuators and energy-efficient-locomotion control schemes for the new generation of legged robots following natural muscle multifunctionality. This multifunctionality is approached by means of merging different technologies (smart materials and conventional technologies) in order to extract the best properties of each one. Some prototypes have already been tested and characterized [27].

This paper presents the development of a biomimetic model of a leg for agile locomotion of quadruped robots. The key principles underlying the superior capabilities of strength, speed, agility and endurance of cursorial mammals, like horses, are analyzed in Section 2 and transferred to technological instantiations in Section 3, where a model of a biomimetic leg for agile locomotion is presented. The proposed concept has been implemented on a real prototype. Section 4 describes the leg design, actuation system and sensorial system. Section 5 describes how variable compliance is achieved at the joints of the leg. Experimental analysis of the leg performance to achieve agile locomotion is analyzed in Section 6, and finally Section 7 presents a discussion on the technological barriers that have been encountered in the technological instantiation of the biomimetic leg model and concludes with some proposals.

## 2. Biological Inspiration for Empowering Robot Legs

**Figure 2.**
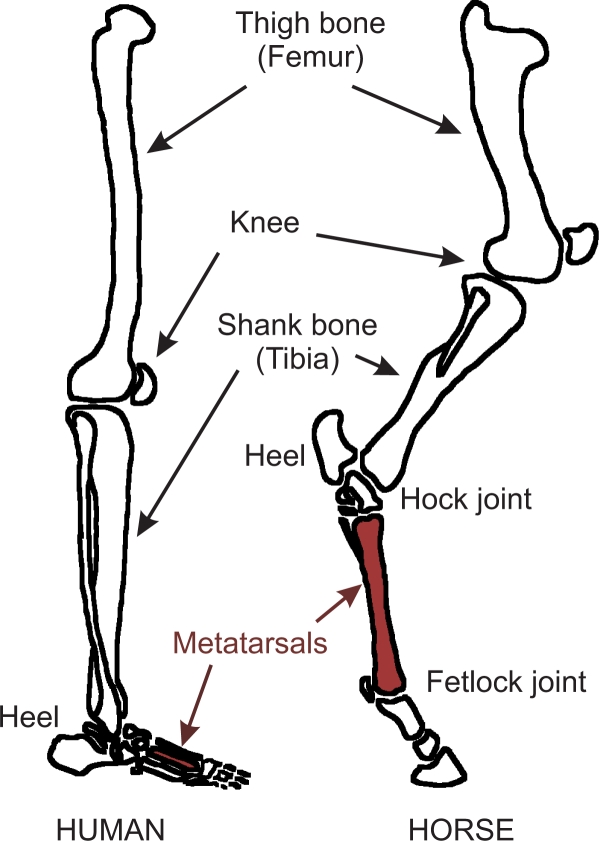
Comparison of horse foot and human foot [28].

As stated above, a quadruped is considered to perform in agile locomotion when it is able to achieve dimensionless speeds up to 0.54 while carrying a payload at least equal to its own weight. In order to design a leg mechanism able to provide the robot with those features, nature is the best source for inspiration. Horse legs are adapted to provide speed, endurance, agility and strength superior to any other animal of equal size [28]. This adaptation is based on longer legs than similar quadrupeds relative to the body size, which provide longer stride lengths. The length of the horse leg is optimal for running, longer legs would probably be difficult to oscillate (giraffes are not able to trot). The cause for the horse relatively long legs is the evolution of the anatomical foot and toe. Horses feet have undergone extensive modification which have enabled these animals to become powerful runners. The most conspicuous change is the reduction of the number of digits: they have retained only one single functional digit. This digit corresponds to the third toe in humans (see Figure 2) and it is able to withstand forces largely superior to those supported by multi-digit toes. Besides, the metatarsal has been so lengthened that it seems more part of the leg than the foot; human metatarsals are located in the arch as shown in Figure 2. Unlike true leg bones, however, it is not directly powered by muscles. Instead, the metatarsal employs spring-like forces from massive ligaments.

The horse rear legs are relatively lightweight, yet strong enough to deliver very large thrusts and to sustain tremendously heavy loads. Again, the leg has evolved to optimize the use of its joints for load bearing. The horse hip joint is mainly a hinge to turn the thigh forward and backward. The abduction/adduction movement is practically negligible [28,29]. Similarly, knee, ankle and fetlock joints (the joint between toe and metatarsal) are 1 DoF joints. Thus, all the muscles and tendons focus their effort in simple joint motions. And all this with enough economy of effort to provide endurance, which is achieved by means of elastic energy storage in tendons during certain phases of the locomotion cycle and the later return of this energy to the more exigent phases.

In the process of copying from nature a desired system performance, one has to be careful in what issues must be extracted and translated to a technological design. The job of the biomimeticist is to identify those elements responsible for producing the desired characteristics on biological systems and to extract the key principles underlying their biological function and then translate them to a technological instantiation that is limited by its own human engineering [30]. One cannot simply copy nature, but rather carefully extract concepts at the level of description that are technically possible to implement. Otherwise, the result of a direct copy would yield a sub-optimal approximation to the desired performance.

When designing powerful robot legs, the engineer could decide to extract the desired characteristics of horse legs which are their superior speed, endurance, agility and strength. In order to translate these characteristics to artificial quadruped legs, the key elements that should be copied have been summarized in Table 2 and enumerated as follows:

1. **Effective leg length** directly affects **speed** and **endurance.** Longer effective leg length improves stride length and consequently leg speed, while longer legs reduce the energetic cost of transport. The average effective leg length of horses is 1.24 m [31] and it represents the 60% of the horizontal horse length from nose to tail [32].
2. **Mass distribution** along the leg determines the natural frequency of leg movement and therefore affects **speed.** It was demonstrated that high speed runner breeds of horses have greater mass located near the hip joint than other breeds. Concretely the 80%–90% of leg mass is located in the thigh in runners. This feature favors a high natural frequency of leg movement and facilitates a higher stride frequency [33]. Added to this, the leg mass relative to body mass influences **agility** of motion. This ratio is between 5% to 8% in horses.
3. **Leg kinematics** influences gait energetics and **endurance**. Movements in the equine limbs occur predominantly in the sagittal plane, which is energetically advantageous in cursorial species [29]. Besides, the use of 1-DOF joints optimize the use of its joints for load bearing, thus improving the **structural strength** of the animal.
4. **Elastic energy storage** in tendons provides **agility** and elastic energy storage, reducing the power requirements at muscles for the more energetic exigent motions and improving **endurance** [34]. The inherent stiffness of tendons also affects the limb natural frequency, which determines the duration of the support phase [35] and consequently influences leg **speed**.
5. **Muscle power** capacity directly determines joint **speed** and limb **strength**.

Taking into consideration these key elements and their role in agile locomotion, a conceptual model of a leg for an agile quadruped has been outlined and its performance has been simulated. This is detailed in the next section.

## 3. Deriving the Biomimetic Leg Concept

The above principles underlying horse power capabilities have been extracted and translated to technological implementation. Firstly, a leg concept which encompasses the key elements has been designed and afterwards, its performance has been analyzed through dynamic simulation.

### 3.1. Effective Leg Length

Taking into consideration that building a quadruped with the size of a horse would be difficult to handle in the laboratory, scaling of the leg length making sure that the effective leg length is the 60% of the body size would comply with the specifications. For a trade-off between reproducing horses dimensions and having a reliable prototype, a scaling factor of 65% has been applied to the design, therefore a robot length of 1.2 m was considered, having an effective leg length of 0.8 m.

### 3.2. Leg Kinematics

The complexity of controlling a planar 4-DoF redundant kinematic chain added to the cost of electronics and actuators and the direct consequence of increasing leg mass as the number of degrees of freedom increases make unfeasible the development of an exact horse-like leg. However, the election of redundant kinematics favors reducing joint torques and thus actuator requirements and power consumption. A possible solution is to use passive elastic elements to drive one or more joints, however, the analysis of joint power requirements for a slow trot (see Section 3.4) advises against purely passive actuation. As a trade-off, a planar 3-DoF leg has been outlined composed of three links: thigh, crus and hoof, connected through 1-DoF joints: the hip, knee and fetlock joints. The lengths of thigh, crus and hoof are proportional to real horse leg’s plus a scaling factor to reach the desired effective leg length, taking into consideration that the use of a 3-DoF model of leg shortens the total leg length in a 34% compared to a horse leg. The 35% reduction in effective leg length plus the increase of 34% in limb length results in a final 1% decrease in each leg link length. Table 3 lists the final link lengths.

**Figure 3.**
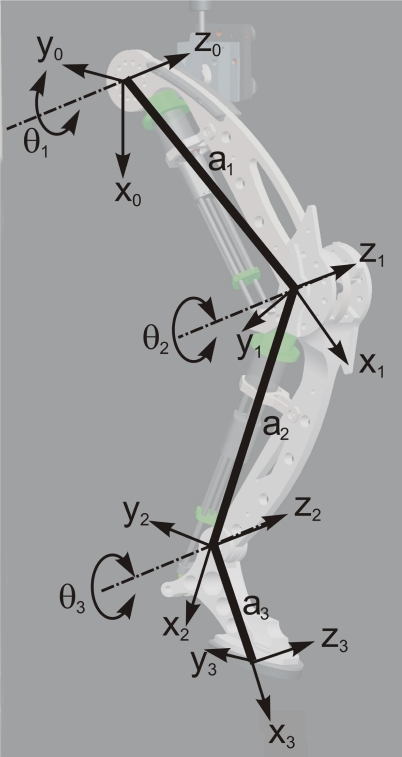
Kinematic model of robot leg.

Figure 3 and Table 4 show Denavit–Hartenberg parameters for the leg kinematics, which correspond to a conventional three-link planar structure. Following this convention, the direct kinematic model provides hoof position and orientation from joint angles as follows:

(2) $$\left[\begin{array}{c} x_{0}\\ y_{0}\\ \varphi \end{array}\right]=\left[\begin{array}{ccc} a_{1}C_{1} & a_{2}C_{12} & a_{3}C_{123}\\ a_{1}S_{1} & a_{2}S_{12} & a_{3}S_{123}\\ \theta_{1} & \theta_{2} & \theta_{3} \end{array}\right]$$

where (*x*_0_*, y*_0_*, ϕ*) are hoof x and y position and orientation respectively in the leg’s base reference frame, and *θ_i_* with *i* = 1..3 are joint angles numbered from hip to fetlock joint. Parameters *a_i_* are the respective link lengths measured as the distance between adjacent joint axes, and correspond to the values listed in Table 3. In Equation (2) *C_i_* and *S_i_* mean cos(*θ_i_*) and sin(*θ_i_*) respectively, while expression *C_ijk_* means cos(*θ_i_* + *θ_j_* + *θ_k_*) and *S_ijk_* means sin(*θ_i_* + *θ_j_* + *θ_k_*).

### 3.3. Mass Distribution

Published work on the experimental determination of equine limb inertial properties show wide ranges of average values for leg segment masses for different horse breeds. Table 5 summarizes average results of an experimental work performed on six Dutch Warmblood horses [36]. Considering that our leg model accounts with three links, the selection of link masses cannot be directly extracted from the biological inertial data, and therefore it was performed in an iterative optimization approach for a later comparison with the average values listed in Table 5. In the optimization approach, the link masses which minimized mechanical power in a locomotion cycle at an average nondimensional leg speed of 0.54 were searched for. The cost function is given by the sum of the mechanical power at the leg joints, given by the product of joint torque and joint speed as follows:

(3) $$C_{W}=\sum_{i=1}^{3}\int_{0}^{T}\tau_{i}(t)\cdot {\dot{\theta }}_{i}(t)dt$$

where joint torque is a nonlinear function of all limb masses *m_i_*, lengths *a_i_*, inertia moments *I_i_* and joint angles, speeds and accelerations, given by the inverse dynamics model of the leg:

(4) $$\tau =D\left(m_{i},a_{i},I_{i},\theta ,\dot{\theta },\ddot{\theta }\right)$$

**Figure 4.**
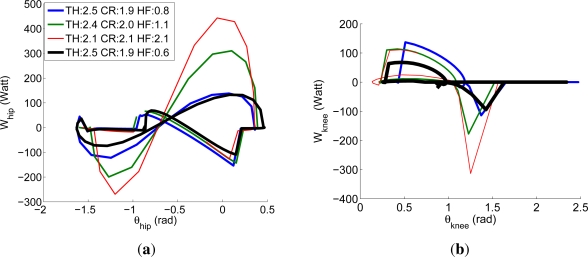
Iterative optimization of leg mass distribution by minimizing the power required at the joints. Convergence is shown in back thicker line for 2.5 kg, 1.9 kg and 0.6 kg at thigh (TH), crus (CR) and hoof (HF) respectively: **(a)** hip power for varying link masses; **(b)** knee power for varying link masses. Initial and intermediate values of the iteration are provided in the figure legend.

Numerically solving the above optimization problem is computationally unavoidable. Therefore, it has been solved by means of an iterative process through dynamic simulation of the leg model using Yobotics! Simulation Construction Set software [37]. This dynamics simulation package was developed at the MIT Leg Laboratory for the analysis of control algorithms in legged locomotion, and it was later commercialized by Yobotics Inc., spin off company from the MIT. The programmed robot simulation provides joint position, speed and torque based on link inertial properties such as mass, center of mass position and inertia tensor by implementing the Featherstone algorithm for deriving the equations of motion. Figure 4 shows results of the iterative process for hip and knee joints, whose variation resulted more significant, and its convergence for the final optimal leg mass distribution.

After iteration, the resulting leg mass distribution was 2.5 kg, 1.9 kg and 0.6 kg for thigh, crus and hoof respectively, which represent a 50%, 38% and 12% of the final leg weight which results 5 kg. From this results and by comparison with biological mass distribution shown in Table 5 it seems that the mass corresponding to the suppressed metatarsus has been distributed between crus and hoof in order to resemble the inertial properties of horse legs. The resulting mass distribution maintains most of the leg mass in the upper leg segment, as in biological horse legs.

### 3.4. Actuator Power Requirements

The technological counterpart of the mammalian muscle is the joint actuation system. In order to determine actuator requirements for each joint, the leg motion was simulated again using the Yobotics! Simulation Construction Set. In order to reach a nondimensional speed of 0.54 for an effective leg length of 0.8 m, an average leg speed of 1.5 m/s was commanded, following Equation (1), and an additional payload of 12 kg was added over the leg. These 12 kg account for one half of the robot weight, which has been assumed to be around 12 kg, and one half of a 12-kg payload carried by the robot. Therefore, the leg was assumed to move in agile locomotion (nondimensional speed of 0.54, supporting dynamic loads and robot payload-to-weight of 1). The details on the locomotion controller used in the simulation can be found in Reference [38].

**Figure 5.**
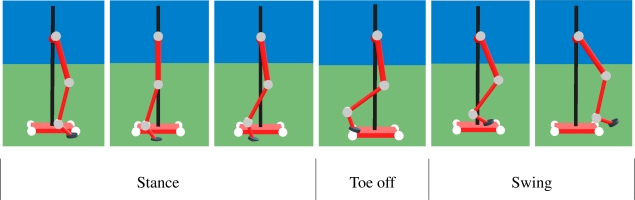
Sequence of the locomotion cycle simulation.

**Figure 6.**
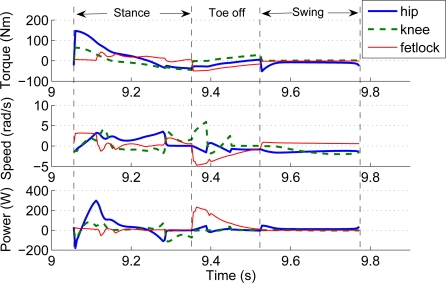
Requirements of torque, speed and power for the joints of a lightweight HADE leg running at 1.5 m/s carrying a 12 kg load in a locomotion cycle.

Figure 5 shows screen-shots of the simulated locomotion cycle. Joint torque, speed and power requirements obtained from the dynamic simulation are shown in Figure 6.

Figure 6 shows a peak demand on hip joint torque near 150 Nm at the beginning of the stance phase. The torque imposed on the hip is mainly used for supporting the robot weight and payload and to propel the robot forward in order to achieve the forward speed of 1.5 m/s. Therefore, the hip accelerates the leg structure, demanding an instantaneous mechanical power of 300 Watts. The knee also contributes to this propulsion, however in a less demanding manner. During toe off, the knee and fetlock do most of the work lifting the hoof. The figure shows large joint speeds required for such motion, as the fetlock has a power requirement of 220 Watts. The swing phase of the leg seems to be the less power-demanding motion.

**Figure 7.**
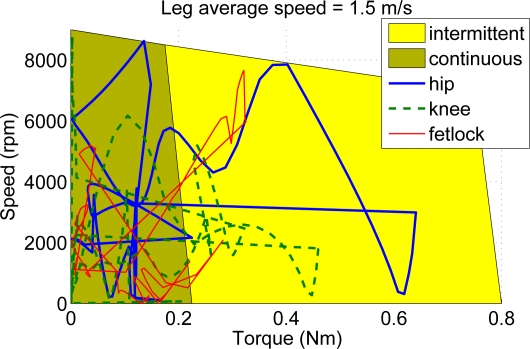
Motor torque-speed diagram overlapped with joint requirements for average nondimensional leg speed of 0.54 considering a biomimetic leg weighing 5 kg.

In order to analyze the suitability of commercially available actuators for the power requirements found, Figure 7 shows the speed-torque diagram of a brushless DC motor by Moog model Silencer BN23-23ZL-03LH. The diagram also shows, overlapped, the joint requirements obtained from the simulation plotted in the form of speed *vs.* torque. The diagram shows that the selected motor fits within the specifications, provided that reduction ratios of 1:290, 1:180 and 1:190 are applied for hip, knee and fetlock joints respectively. Although some parts of the joint trajectories fall within the intermittent operation range of the motor, from Figure 6 we can observe that the duration of the large-power spans is almost instantaneous, lasting less than 50 milliseconds, which is supported by the intermittent motor operation. Therefore, the selected motor is suitable for the application in terms of power requirements. Besides, a light weight (0.5 kg) and a compact design provide suitable power-to-weight and power-to-volume ratios.

### 3.5. Elastic Energy Storage

In biological quadrupeds energy is stored in tendons during some phases of the locomotion cycle and later released to provide kinetic energy to the leg in those phases which demand a larger power consumption. In a horse, this energetic conversion occurs in several parts of its body, however here we will concentrate on the use of elastic recoil in tendons of the hind legs, which is indeed the principal source of energy in horses. The flexing and then straightening of the hind legs is the mechanism that first stores and then releases energy making use of the tendons. In a horse, energy is stored in the musculotendinous system during the first half of the support phase by stretching of the viscoelastic connective tissues. During the second half of support much of the stored energy is recovered as the connective tissues shorten [39].

**Figure 8.**
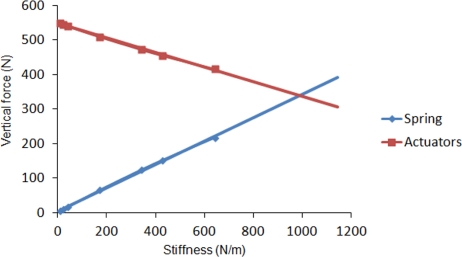
Effect of spring stiffness on vertical propulsive force exerted by the actuators at the hip, knee and fetlock joints.

**Figure 9.**
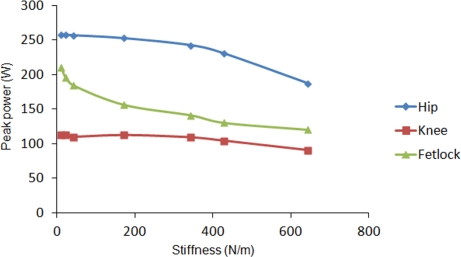
Effect of spring stiffness on the power required at the hip, knee and fetlock joints.

**Figure 10.**
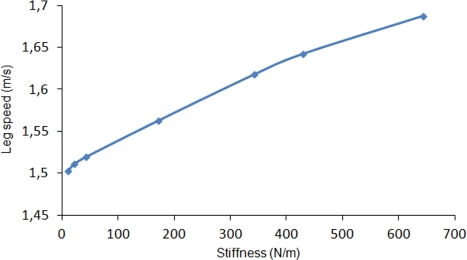
Effect of spring stiffness on the duration of the support phase.

In order to analyze the energetic advantage of introducing elastic energy recovery in the robot leg, a spring has been attached to the simulated leg from hip to fetlock joint. The leg motion is again commanded at an average leg speed of 1.5 m/s. The spring stiffness is increased in each cycle. Figure 8 shows the effect of increasing spring stiffness on the vertical propulsion force exerted by the actuators on the leg’s base reference frame. Although simulations were performed for spring stiffness varying up to 650 N/m, a linear tendency line has been plotted in order to show the behavior for larger stiffness. As it can be observed, as long as the spring stiffness increases, the vertical force exerted by the spring increases, what results in a lower force demand provided by the actuators. The tendency lines cross at a spring stiffness of 980 N/m and for larger stiffness the propulsion force exerted by the spring exceeds the propulsion force required by the actuators. Figure 9 shows how the peak power requirement at each joint diminishes as the spring stiffness increases. It also shows a larger dependency on spring stiffness at hip and fetlock, while the knee power shows a minor variation. For the maximum simulated spring stiffness of 650 N/m a 31% of power is reduced due to the elastic energy recovery. However, it is worthwhile noting that the spring stiffness should not be increased too much, because a too large stiffness would dominate the leg dynamics, neglecting the action of the actuators thus yielding a pure spring-mass system without possibility of adaptation to the environment. The effect of leg elasticity on leg speed is shown in Figure 10. In this figure it can be observed that, although a leg speed of 1.5 m/s is commanded, the increase in leg stiffness produces a progressive rise in leg speed by reducing the support time.

As larger values of spring stiffness would dominate the leg dynamics interfering in the locomotion controller, for this biomimetic leg model we have considered a spring stiffness of 650 N/m as a trade-off between leg control performance and power efficiency by reducing the power consumption in a 31%.

Following the biomimetic leg concept herein presented, a real leg prototype has been designed, developed and tested. Section 4 presents the design process and final prototype, while the following sections will go in deep into the experimental locomotion. Conclusions on the effectiveness of translating biomimetic features to robot prototypes are finally given.

## 4. The HADE Leg for Agile Locomotion

**Figure 11.**
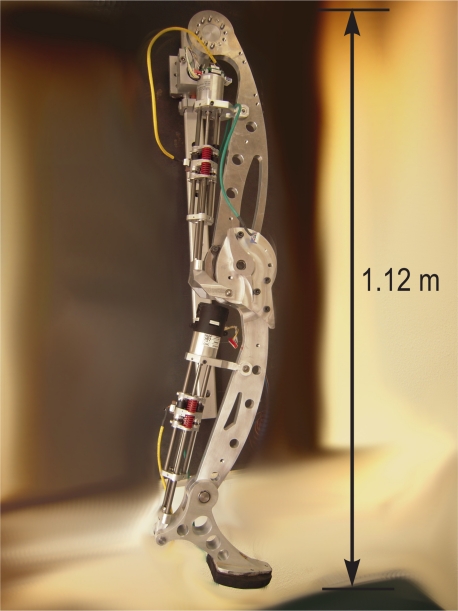
First prototype of the HADE Leg resembling a horse leg.

The HADE leg has been designed following the valuable guidelines obtained from biomimetism: it is a relatively long leg with three 1-DoF joints, featuring a mass distribution which reduces link mass for distal links, and which is propelled by series-elastic actuation. Figure 11 shows a picture of the HADE leg which resembles the anatomy of a horse rear leg. The rest of this section will develop on the details of leg design, kinematics and the actuation system selection.

### 4.1. Prototype Design and Manufacturing

**Figure 12.**
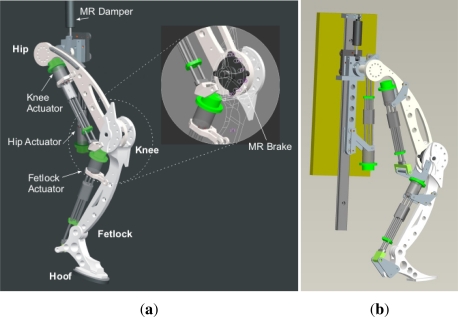
Design of first prototype of the HADE Leg. **(a)** The MR rotary damper at the knee is showed in zoom-in view; **(b)** reverse view of the actuators.

The design of the first prototype of the HADE leg is shown in Figure 12. It is a planar 3-DoF leg composed of three links: thigh, shank (including metatarsal) and hoof, connected through the hip, knee and fetlock joints. The mechanical structure of each link has been designed in order to achieve large payload-to-weight ratio and provide impact tolerance. The hoof is endowed with a rubber pad which provides shock absorption and damping at ground contact and also increases friction between foot and ground improving horizontal propulsion.

The large payload-to-weight requirements of the structure could be reached by manufacturing the mechanical structure using Alumec 89, a high strength aluminium alloy which undergoes a special cold stretching operation for maximum stress relief. This material is being used in the aerospace industry and it shows the best payload to weight properties. Unfortunately, these special alloys are only at the reach of big companies of the aerospace industry and it was not possible to obtain the Alumec 89 for the manufacturing of a robotic leg prototype. Therefore, for the purpose of testing the performance of the biomimetic leg concept, the leg has been manufactured in Aluminium 7075, obviously keeping in mind the increase in weight that the final prototype will suffer due to the different material mechanical properties. Table 6 compares mechanical properties for Aluminium 7075 and Alumec89.

Table 7 shows link dimensions and masses of the first prototype. The position of center of mass and inertia tensor are referred to the joint reference frame following the Denavit–Hartenberg convention as shown in Figure 3. Joint ranges of motion and leg kinematics have been detailed in the Appendix.

### 4.2. Actuation System

In order to select the actuators for the HADE leg, the observed joint requirements were transferred back to the motors taking the transmission system into account.

#### Actuator Requirements

**Figure 13.**
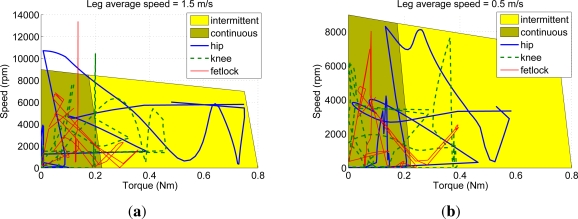
Motor torque-speed diagram and joint requirements for the real leg prototype manufactured in Aluminium 7075 **(a)** average leg speed of 1.5 m/s; **(b)** average leg speed of 0.5 m/s.

Due to the different link masses that the leg prototype features compared to the biomimetic leg concept, actuator requirements have been determined by modifying the simulation with the real prototype inertial parameters. In order to determine motor requirements, joint speed-torque trajectories obtained from the simulated locomotion cycle have been plotted over the motor characteristic diagram, considering actuator efficiency and the transmission reduction ratios, which are detailed in the Appendix. These plots are shown in Figure 13.

The requirements of low actuator weight, actuator power over 400 Watts (considering an electro-mechanical efficiency of 90%), compactness and large speed are all met by brushless DC motors. Figure 13(a) shows the speed-torque diagram of the Moog Silencer BN23-23ZL-03LH. The diagram also shows joint requirements based on the speed-torque trajectories obtained from simulation of the real prototype running at 1.5 m/s. It can be observed how all three joint trajectories fall outside from the motor operational region. Although knee and fetlock could cope with having lower reduction ratios, there is no way to meet the hip requirements using the selected motor. Unfortunately, selecting a larger motor in power would yield a extra weight and size, which will again increase the link masses so we finally decided to implement the actuation system based on the initially selected DC brushless motor, at a cost of speed reduction. Figure 13(b) shows the speed-torque diagram of the same motor for the real leg moving at 0.5 m/s so that the selected actuators can provide leg motion.

The requirement of including elasticity into the actuation system added to the need for a precise force control scheme led us to use Series Elastic Actuators (SEAs). Specifically, the Yobotics’ SEA23-23 were selected. The next paragraphs will explain in detail this actuator and the effect of series elasticity.

#### Series-Elastic Actuation

**Figure 14.**
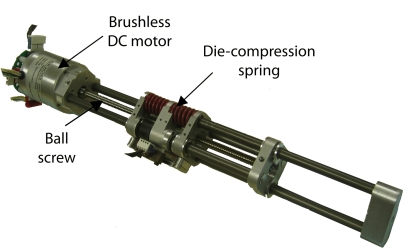
Yobotics SEA23-23.

**Figure 15.**
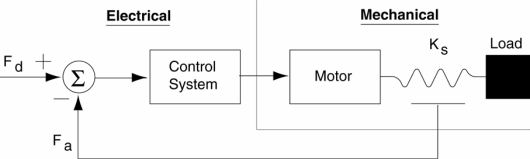
Schematic diagram of a Series Elastic Actuator [40].

Added to the power requirements described above, the desired elastic behavior of the actuation system can be met by using series elasticity between the motor and the joint. The HADE leg uses Series Elastic Actuators (SEA), a family of actuators specifically designed for the force control of robotic systems. They were designed at the MIT Leg Laboratory and they are currently commercialized by Yobotics Inc. They are backdriveable, compliant actuators with resistance to impact and vibration. The power-to-weight ratio of this actuator in intermittent operation is near 600 W/kg, which fits within the HADE leg requirements. SEAs are low motion, high force-to-weight, high power-to-weight actuators, which employ a novel mechanical design architecture that goes against the common machine design principle of “stiffer is better” [40]. They are composed of a motor and a transmission to the load, but they have an elastic element connected in series between the transmission and the load. Figure 14 shows a photograph of the Yobotics SEA23-23 and Figure 15 shows a simple diagram of a general SEA.

In SEAs, stiff load cells are replaced with a compliant element, a spring, thereby increasing the robustness and stability and lowering the cost. The spring allows us to indirectly measure joint forces by measuring the deflection of the spring. This means that it is actually a transducer. In addition, the elastic element gives the actuator low output mechanical impedance, in contrast with traditional actuators with high power-to-weight ratio which usually have high output impedance.

The motor used in SEAs features good position accuracy to give a good force output. The better the motor can modulate the spring position, the cleaner the force output of the spring. Besides, the elastic element filters noise and facilitates the increase of the force-controller gain within stable operation. As a whole, SEAs improve conventional actuators in force control response.

All actuation requirements stated throughout this section are met by the Yobotics SEA23-23 shown in Figure 14 for hip and fetlock joints. Knee requirements are met by the same actuator using a 5-mm lead ball screw. Table 8 lists actuator specifications for the three joints of the HADE leg.

### 4.3. Sensorial System

The state of the HADE leg system is monitored by means of torque and position measurements at each joint, and the measurement of ground-reaction forces. Joint torques and angular positions are measured by force and position sensors respectively, which are enclosed in each SEA, while the ground-reaction force is acquired by a force sensor placed inside the pad of the hoof. The force provided by the actuators is measured using linear encoders which measure the spring deflection, thus computing the force on the load using Hook’s law. The position sensors at the actuators are also linear encoders that measure the displacement of the shaft. Specifications of these encoders are listed in Table 8.

**Figure 16.**
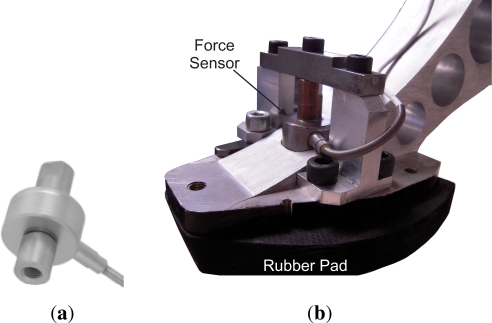
**(a)** Honeywell precision miniature load cell; **(b)** Sensor mounted on the hoof of the HADE leg.

The force sensor at the pad to measure ground-reaction forces is a Honeywell miniature precision load cell model 34 (P/N AL312CR). This sensor is placed inside the pad (see Figure 16) to measure vertical ground forces ranging from 9.8 N to 2200 N. The sensor signal is acquired by a 16-bit A/D converter which provides a resolution of 2.10^−13^ *N*. High accuracies of 0.15%–0.25% full scale are achieved. Residual effects of off-axis loads are minimized.

**Figure 17.**
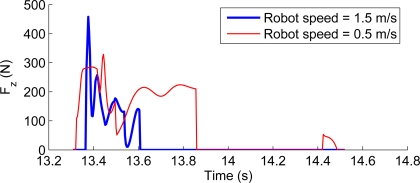
Ground reaction forces for the leg walking at 1.5 m/s (biomimetic design) and walking at 0.5 m/s for the real leg prototype.

Vertical ground reaction forces are used by the locomotion controller to maintain a desired ground contact force during the stance phase. Figure 17 shows vertical ground forces at the hoof during one locomotion cycle for the leg walking at 0.5 m/s and compares them with the simulated forces for the robot walking at 1.5 m/s assuming a lightweight leg design. The figure shows a sharp impact to the ground at the beginning of the cycle coinciding with the beginning of the stance phase. This impact seems to soften at lower speeds, such as 0.5 m/s although larger loads are involved considering the increase in link weight in the current leg prototype.

## 5. Controlling Joint Compliance

### 5.1. Active Joint Compliance

**Figure 18.**
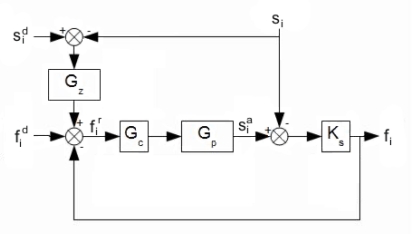
SEA compliance control scheme block diagram.

**Figure 19.**
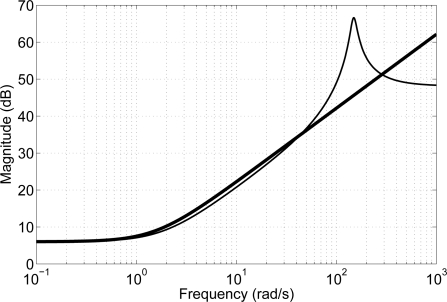
Bode diagram of the actuator impedance (thin line) overlapped with the ideal spring-damper system (thick line).

Series elasticity provides good force control at the joints and adds some shock tolerance to the mechanism. Also, it decouples the motor inertia from the load, so that slight position errors will be absorbed by the elastic element, preventing the control system from reacting to unexpected impacts. A compliance controller programmed in the SEA imitates a desired dynamic behavior inside the actuator response bandwidth. As in this case the controlled actuator bandwidth is 35 Hz, then in the range 0–35 Hz the controller allows the actuator to behave like a spring-damper. The block diagram of the compliance control scheme is shown in Figure 18, where *G_z_* is the model corresponding to a spring-damper system, *G_c_* is the transfer function of the inner loop controller, *G_p_* is the actuator’s transfer function, *K_s_* is the die compression spring constant, $f_{i}^{d}$, *f_i_* and $f_{i}^{r}$ are the desired force, exerted force, and the force reference respectively. Finally, $s_{i}^{d}$, *s_i_* and $s_{i}^{a}$ are the desired position, the load position and the actuator position. Choosing *G_z_* = *K* (1 + *bs*) and by modifying the desired spring-damper coefficients *K* and *b*, the mechanical impedance is modified and it can be adapted to different leg velocities and ground stiffness. Figure 19 shows a Bode diagram of the system behavior (thin line) overlapped with an ideal spring damper (thick line) system, which shows that for frequencies below the controlled actuator bandwidth (35 Hz), the controlled actuator behaves like a spring-damper. These properties largely improve the adaptation to the ground in high-speed locomotion.

**Figure 20.**
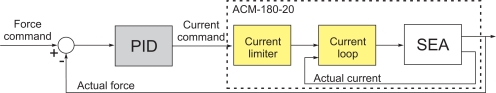
Joint force control based on a PID.

The inner force controller in Figure 18 is based on conventional PID control acting directly on the current command of the SEA’s power amplifier. Figure 20 shows a block diagram of the inner force control scheme.

### 5.2. Controllable Passive Damping at the Knee

Active damping is specially required at the knee in order to achieve a natural motion [41]. Such active damping could be provided by means of active compliance control of the actuators, however, at a cost of energy consumption considering that the knee is mostly dissipating power during the gait cycle. However, it is strongly required to reduce the energy consumed by the actuators during the damping motion in these applications where energy efficiency is a primary goal. Therefore, the use of a passive damping device of controllable damping coefficient is desirable.

**Figure 21.**
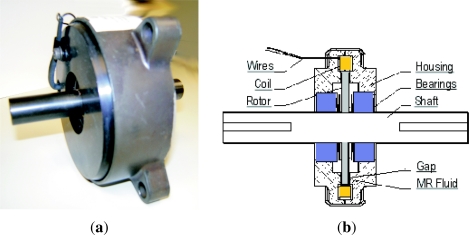
**(a)** LORD RD-2087-01 Magneto-Rheological rotary damper; **(b)** mounting scheme.

**Figure 22.**
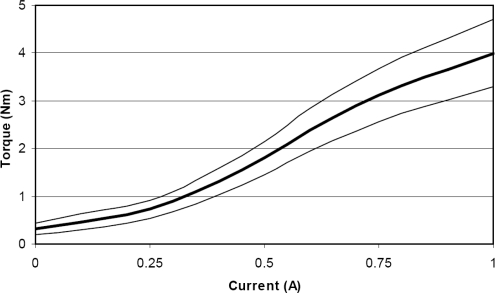
Typical torque-current curve of the LORD RD-2087-01 MR rotary damper.

In this project a LORD RD-2087-01 Magneto-Rheological (MR) rotary damper has been placed at the knee to test the efficiency of actively controlling knee damping along the gait cycle (see Figure 21(a)). Magneto-rheological brakes and dampers are resistive actuators based on Magneto-Rheological Fluids, a kind of smart material of the Magnetoactive family [26]. The MR rotary damper is designed as shown in Figure 21(b): An outer housing attached to the thigh contains a rotor joined to the knee axis. The MR fluid fills the space between the rotor and the outer housing. There is a coil attached to the housing, leaving a small space for the MR fluid between the rotor and the coil. As electric current circulates by the coil, a magnetic field is applied to the MR fluid, which yields a significant change in the rheologic characteristics of the MR fluid which is due to the alignment of the ferromagnetic particles of the MR fluid into chains. As a result, an increased shear stress results between rotor and housing, and consequently an increased resistive torque output is achieved in less than 10^−2^ s response time. The increase of torque that damps the rotor depends directly on the magnetic field applied and increases linearly with rotor speed, thus providing controllable viscous rotary damping. A detailed background on the principle of operation of Magneto-rheological Fluid (MRF) devices can be found in Reference [42]. Figure 12 shows the integration of the MR rotary damper inside the knee of the HADE leg. The maximum power consumption of the MR rotary damper is 1 A at 12 VDC for a maximum resistive torque of 4 Nm. Figure 22 shows the typical torque-current curve for the MR rotary damper provided by the manufacturer.

**Figure 23.**
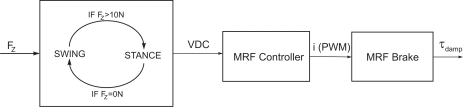
Block diagram of the MRF damper control scheme for passive knee damping.

Using the MR rotary damper, the SEAs are used for active motion, while passive damping is performed by the MR rotary damper. The MR rotary damper is commanded through a device controller kit provided by the MRF damper manufacturer. This controller device provides closed loop current control to compensate for changing electrical loads up to the limits of the power supply. The controller device is operated as an interface device for computer control of the MRF damper. When a continuous 0–5 VDC signal is sent to the controller device it commands a proportional pulse-width modulated current signal, where 0% duty cycle matches 0 A and 100% duty cycle matches 1A. A finite-state machine switches between stance and swing states and changes the voltage input to the controller device which in turn modifies the knee damping coefficient. The finite state machine switches from stance to swing when the foot/ground reaction force sensed becomes 0 N and returns to stance state when the ground reaction force reaches 10 N. A block diagram of the MRF control scheme is shown in Figure 23.

The controller gains have been tuned manually to achieve a natural-looking motion, which are 1.45 Nm rad^−1^ s during the support phase and 0.9 Nm rad^−1^ s during the leg swing phase.

## 6. Experimental Locomotion

In order to test the performance of the HADE leg for agile locomotion, experiments showing the speed and loading capabilities have been conducted.

A first experiment has been performed with the leg running on the air. The leg motion is conducted by the locomotion controller detailed in Reference [38], which is based on a state machine that commands the three independent joint controllers, one for each joint, already described in Section 5. The MRF damper controller in Section 5 is used at the knee during swing to damp the shank inertia.

**Figure 24.**
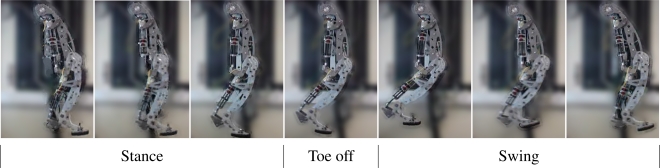
Sequence of the locomotion cycle of the HADE leg.

**Figure 25.**
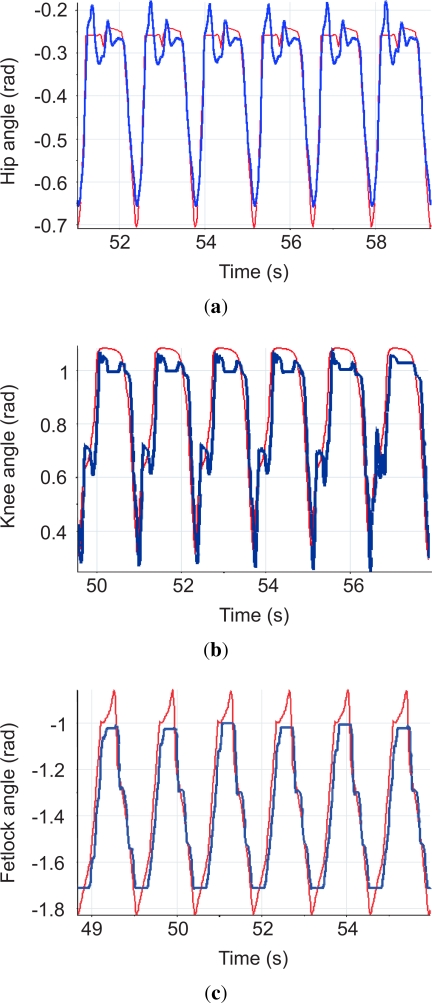
Experimental joint trajectories (thick line) and simulated joint trajectories (thin line) **(a)** hip; **(b)** knee; **(c)** fetlock.

Figure 24 shows snapshots of the locomotion cycle. The video of this experiment can be found at [8]. Figure 25 shows hip, knee and fetlock joint trajectories during six locomotion cycles following the reference trajectories given by the gait controller. The maximum cycle speed achieved in this experiment was 0.6 m/s, with a duration of leg cycle of 1.2 s, half for swing and half for stance phases. These experiments have been performed with the leg running in the air, and the ability of the system to tackle with leg dynamics during agile locomotion has been validated.

**Figure 26.**
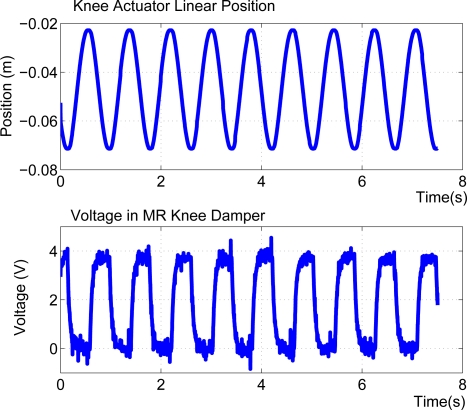
Experimental trajectories of the knee actuator and power consumption of the knee MRF damper during cyclic flexion-extension motions of the leg in support carrying a 12-kg payload.

**Figure 27.**
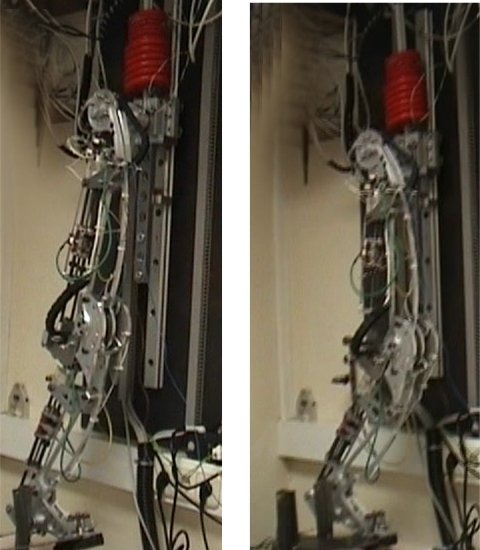
Experimental cycles of knee flexion-extension with a 13-kg payload over the hip.

A second experiment is conducted with the leg in support phase and it has been commanded to run knee flexion-extension cycles at increasing frequency while carrying a 13-kg payload over the hip joint. The HADE leg was able to run at 1.2 cycles per second while carrying a payload of 13 kg. This frequency provides the minimum duration of the stance phase, 0.83 s. Added to the duration of leg swing obtained in the previous experiment, the minimum total cycle time when carrying a 13-kg payload is 1.33 s. The stride length is 0.7 m which yields a leg maximum average speed of 0.54 m/s. Figure 26 shows the activation and deactivation of the MRF damper during the cyclic motion. The video of this experiment can be found at [8] and two snapshots corresponding to knee extension and knee flexion respectively are shown in Figure 27.

The final leg speed achieved (0.54 m/s) is slightly larger that the initially estimated during the analysis of actuation system specifications (0.5 m/s). This slight improvement could be due to the elastic recoil in SEAs. The spring stiffness of the SEA23-23 is 3 · 10^5^ N/m, and the spring deflects a maximum of 7 mm, which results in a maximum potential energy of 9.5 J that is transferred to the joint as kinetic energy, providing an added speed about 0.04 m/s to the leg. Nevertheless, this amount of elastic energy recovered is still far from the 31% of elastic energy specified in our biomimetic leg concept.

## 7. Discussion and Conclusions

Traditionally, the design of legged robots has encountered its inspiration in engineering principles and experience taken from conventional industrial robotics. Low-performance legged robots have been the result of imposing precision trajectory following in the joints of stiff legs, actuated by conventional motors. The success of the new generation of legged robots, aimed at featuring endurance, power and agility similar to their biological counterparts, could find the key on biomimetic designs and gait controllers.

This work has developed a concept of agile-robot leg based on the extraction of those key principles that play a significant role in the agility, power, speed and endurance of biological quadrupeds. Concretely, this work has found inspiration in horse legs because of their superior characteristics for agile locomotion. Dynamic simulation of a leg model that joins the key factors extracted showed a potential improvement in artificial agile legged locomotion.

However, this paper has shown that it is not only a matter of extracting the key principles of agile locomotion from nature. In fact, the success of the development of efficient biomimetic prototypes strongly depends on the improvement of technological key factors that currently limit the performance of biomimetic designs. In this paper, a real prototype of a biomimetic leg has been presented. Difficulties in engineering the biomimetic key principles conducted to a final prototype not able to achieve the final goals for agile locomotion: robot’s nondimensional speed over 0.54 and payload to weight ratio between 1 and 1.5. Although the payload capacity has been tested, the leg was not able to walk faster than 0.54 m/s, which results in a non-dimensional speed of 0.2, almost three times lower than the initial goal. The cause of this failure is mainly the use of a structural material that did not meet the initial specifications, or the need for actuators featuring larger power-to-weight and power-to-volume ratios.

This paper puts some light into the technological challenges that require an impulse in research in order to develop fully functional agile legs. These key factors are:

1. Development of strong and lightweight materials and structures, available for the research community, not only at the reach of big companies.
2. Specific actuators featuring large specific power and torque, and large power density.
3. Inherent compliance in the structure or in the actuators. The series elasticity that current SEAs exhibit results not enough for elastic recoil purposes. The spring in SEAs in used as a force sensor, and for this reason, very small deflections and a very large stiffness is required if it is required to measure large forces. Therefore, the structural elasticity should be included as part of the structure, as viscoelastic tendons. The larger the deflection of the tendons, the larger the potential elastic energy recovery.

Besides the identification of technical factors that need to be improved, this paper has shown the effectiveness of a hybrid, joint actuation system based on series elasticity and MR dampers which has been tested to achieve a compliant, natural motion in an energy efficient fashion. This combination of SEA and MR damping has been tested in a real leg prototype, the HADE leg. In the inner control loop of joints, force control strategies have been performed in order to provide compliant joint motion, which will improve structural robustness and offer safe interaction with the environment.

## Figures and Tables

**Table 1.** Performance of significant quadruped robots developed in the last 12 years.

**Robot**	**Supports dynamic loads**	**Payload/Weight**	**Max. dimensionless speed**	**Year**
Aibo [9]	No	0	0.35	1999
Scout II [10]	Yes	0.02	1.17	1999
SILO4 [11]	No	0.59	0.06	2002
TITAN XI [12]	No	0.06	0.003	2002
Tekken II [13]	Yes	0	0.65	2003
LittleDog [14]	No	0 *^a^*	0.23	2005
Tekken 3 & 4 [15]	Yes	0.2	0.65	2005
Kolt [16]	Yes	–	0.35	2005
Rush [17]	Yes	0	0.64	2007
BigDog [4]	Yes	1.41	0.72	2008 *^b^*

^a^ LittleDog’s complex computing is provided by an off-board processor, it is not a self-contained autonomous quadruped [2];

^b^ Although the first BigDog robot was developed in 2005, this data corresponds to the 2008 BigDog prototype.

**Table 2.** Key elements and their influence on desired characteristics of horse legs.

	**Speed**	**Endurance**	**Agility**	**Strength**
Effective length	✓	✓		
Mass distribution	✓		✓	
Kinematics		✓		✓
Elasticity	✓	✓	✓	
Muscle power	✓			✓

**Table 3.** Characteristic robot lengths (in meters) based on biomimetism.

**Body**	**Effective leg**	**Thigh**	**Crus**	**Hoof**
1.3	0.8	0.4	0.36	0.19

**Table 4.** Denavit–Hartenberg parameters of the leg model.

**Joint**	***a****_i_*	***d****_i_*	***α****_i_*	*θ_i_*
**Hip (1)**	*a*_1_	0	0	*θ*_1_
**Knee (2)**	*a*_2_	0	0	*θ*_2_
**Fetlock (3)**	*a*_3_	0	0	*θ*_3_

**Table 5.** Experimental average values of horse leg segment masses expressed in kilograms, extracted from related literature [36], and compared to final results of an iterative search performed through simulation of a 3-DoF leg; percentages of segment mass relative to leg mass are given inside brackets.

	**Thigh**	**Crus**	**Metatarsus**	**Hoof**
Dutch Warmblood horses	2.1 (59.6)	0.96 (26.5)	0.39 (10.8)	0.1 (2.9)
Results for 3-DoF leg	2.5 (50)	1.9 (38)	–	0.6 (12)

**Table 6.** Mechanical properties of Aluminium alloys.

	**AA 7075**	**ALUMEC 89**
**Ultimate Tensile Strength** (MPa)	540	590
**Yield Strength** (MPa)	480	550
**Young’s Modulus** (*N/mm*^2^)	70,000	71,500
**Density** (*kg/dm*^3^)	2.8	2.8

**Table 7.** Physical parameters of the HADE leg prototype.

**Link**		**Thigh**	**Shank**	**Hoof**
**Length (mm)**		505	461	205

**Mass (kg)**		4.6	3.7	1.6

**Center of mass***^a^***(mm)**	x	325	143	93.2
	y	−10.8	−16.6	−41.1
	z	35.2	10.1	−0.02

**Inertia Tensor***^a^* (10^−3^**Kgm**^2^)	*I_xx_*	16.4	8.95	5.17
	*I_xy_*	−1.87	1.33	2.61
	*I_xz_*	−0.05	−0.52	0.001
	*I_yy_*	1060	110.7	9.36
	*I_yz_*	0	−0.01	−0.001
	*I_zz_*	1050	108.6	13.48

^a^ CM position and Inertia tensor refer to Denavit-Hartenberg joint reference frames.

**Table 8.** Actuator specifications.

**Joint**	**Hip/Fetlock**	**Knee**
**Model**	SEA23-23	SEA23-23 (modif.)
**Weight (kg)**	1.1	
**Diameter (mm)**	58	
**Length (mm)**	150 + stroke	
**Maximum Stroke (mm)**	150	
**Ball-screw pitch (mm)**	2	5
**Motor**	Moog Silencer BN23-23ZL-03LH	
**Rotor inertia (gcm**^2^**)**	106	
**Operating voltage (V)**	48	
**Maximum current (A)**	20	
**Maximum speed (m/s)**	0.27	0.67
**Cont. force at max. speed (N)**	566	226.4
**Continuous power (W)**	166	166
**Int. force at max. speed (N)**	1,300	520
**Intermittent power (W)**	629	
**Int. Power to weight (W/kg)**	571	
**Closed-loop bandwidth (Hz)**	35	
**Saturation bandwidth (Hz)**	7.5	
**Efficiency (%)**	90	
**Spring**	Century Die Spring 1222-A	
**Spring stiffness (N/mm)**	4 × 78.4	
**Encoder**	USDigital EM1-0-500	
**Pos. resolution (lines/mm)**	19.68	
